# Study protocol: a randomised, controlled trial of a nurse navigator program for the management of hepatitis C virus in patients with severe mental disorder

**DOI:** 10.1186/s12912-022-00870-w

**Published:** 2022-04-20

**Authors:** Casta Quemada-González, José Miguel Morales-Asencio, María M. Hurtado, Celia Martí-García

**Affiliations:** 1 Mental Health Unit, Regional University Hospital, Instituto de Investigación Biomédica de Málaga (IBIMA), 29009 Málaga, Spain; 2 Faculty of Health Sciences, Universidad de Málaga, Instituto de Investigación Biomédica de Málaga (IBIMA), Málaga, Spain; 3 Faculty of Health Sciences, Universidad de Málaga, Málaga, Spain

**Keywords:** Hepatitis C, Mental disorders, Critical pathways, Patient care planning, Nursing

## Abstract

**Background:**

To evaluate the impact of a nurse navigation program on treatment adherence and resolution of hepatitis C infection in patients with severe mental disorder.

**Methods:**

An open, randomized, controlled trial with blinded outcome assessment. The intervention group will engage in a nurse navigation program designed by mental health nurses. The program involves active screening for patients with severe mental disorder. The patients and caregivers included in the program will receive information, training, support and guidance throughout the treatment and recovery process, which involves different healthcare professionals and units. The control group will receive the standard of care, which includes follow-up by a family physician, referral to the hepatologist, serological testing, new referral to the hepatologist, onset of treatment, and follow-up. Multidisciplinary care will be provided along a coordinated and seamless clinical pathway led by a nurse navigator.

The primary endpoints are total recovery (hepatitis C cure) and treatment adherence. Occurrence of symptoms of schizophrenia and health-related quality of life will be also recorded. Follow-up of patients will be performed three and six months after the administration of antiviral treatment.

The study was authorised by the Ethics Committee of Malaga in December 2021. Funding was approved in March 2021.

**Discussion:**

If this intervention is proven to be effective in improving treatment access and adherence, it will represent a step forward in addressing a chronic health issue that is 16 times more prevalent in the population with severe mental disease. Finally, this intervention may lead to the detection of undertreated HCV infection in this population of patients.

**Trial registration:**

This protocol has been registered in ClinicalTrials.gov with identifier code NCT04891445 on May 18, 2021.

## Introduction

Patients with severe mental disorder (SMD) (schizophrenia, bipolar disorder, schizoaffective disorder, major depression, and others) often experience poorer physical health than the general population. This may be explained by internal factors such as a sedentary lifestyle, poor diet and drug uses [1], which lead them to die, on average, 15–20 years earlier than the general population [2] Suicide rates in this population are also higher than in the general population [3].

This group is also at a higher risk of acquiring infectious diseases such as human immunodeficiency virus (HIV), hepatitis B (HBV), or hepatitis C (HCV), because they are more prone to engage in risk behaviors [4]. Attention needs to be paid on HCV, as this virus affects the liver, may cause a chronic disease, and may be transmitted to other people [5]. Moreover, Hepatitis C can be cured whereas Hepatitis B and HIV not.

The focus must also be placed on other healthcare issues, such as the lack of programs for the prevention, identification, and treatment of physical diseases in patients with SMD. A recent study in Ethiopia revealed that the prevalence of HCV infection (1.29%) is higher in patients with SMD, although it most frequently remains undiagnosed in this population (3 of 4) [6]. A high percentage of patients remain asymptomatic, the virus is associated with a higher risk of mortality, and patients with SMD frequently have treatment adherence problems [7]; therefore, the identification and proper management of the disease in SMD patients emerges as a highly relevant issue.

Some studies have shown how mental health nurses can adopt effective strategies to improve treatment adherence in patients with SMD [8, 9].

### Background

The scientific literature shows a well-established association between the presence of SMD, such as schizophrenia, and poor physical health, especially in young people [10]. As compared to the general population, life expectancy in women and men with schizophrenia is 15 and 20 years lower, respectively [2]. Around a fifth of premature deaths are associated with suicide and accidents, but most are caused by physical illnesses [11], including, among others, infectious diseases such as HIV and hepatitis [12, 13]. Moreover, the risk of death from infectious disease is almost ten times higher in people with psychosis and schizophrenia, than in the general population [14].

There is evidence that patients with SMD are at an increased risk for HIV, HBV and HCV infection, as compared to the general population [15]. Generally, patients with a psychiatric illness are less aware of the transmission of infectious diseases and protective measures [16]. Risk sexual behaviours, low quality of life, or long-term hospitalization may contribute to higher infection rates in this population [15].

The prevalence of HCV infection among psychiatric patients has not been clearly established in Spain. Previous studies report a prevalence of HCV 2 to 16 times higher in SMD patients than in the general population [17–19].

Although HCV remains virtually asymptomatic in many cases, patients with chronic infection experience a variety of symptoms that include depression, fatigue, and neurocognitive deficit, which affect their quality of life [20]. Likewise, patients with SMD are generally on treatment with neuroleptic or stabilizer drugs, which cause dejection, tiredness, and anhedonia. In addition, since SMD causes similar symptoms, this symptomatology will be rarely attributed to a physical health problem such as HCV [12].

As a result, HCV is frequently underdiagnosed and untreated in patients with SMD [6], mainly due to the side effects of interferon, the only treatment available until a few years ago. Several clinical studies reveal that immunomodulatory treatment with interferon causes numerous physical and mental side effects, mainly symptoms of depression, anxiety, psychosis, and other non-specific symptoms such as fatigue, irritability, sleep disturbances, psychomotor retardation, decreased libido, insomnia, and/or cognitive impairment [21–25]. These side effects cause this population to poorly tolerate HCV treatment.

In the recent years, Direct Acting Antiviral therapy with proven efficacy in the general population and very limited side effects have been approved [26]. These new treatments offer the possibility of re-evaluating the SMD population for administering the new treatments to the patients who did not tolerate previous treatments.

Protocols of mental health services do not usually include routine screening for HCV, which may remain underdiagnosed and untreated [6]. In addition, HCV infection may remain active in SMD patients [21].

Nielsen et al. proposed the implementation of programs for the management of mental and physical illnesses in SMD patients coordinated by a multidisciplinary team. The purpose of these programs is to optimize treatment outcomes and reduce dropout rates [27]. Most collaborative management programs have been endorsed by associations related to mental health, diabetes, and cardiology [28].

Several studies have analysed the effectiveness of mental health nurse-led interventions in addressing many of the challenges that standard management models are unable to address. Thus, forty clinical trials evaluating nurse-led multifaceted interventions have been identified in a scoping review; many of them were aimed at assessing treatment adherence and medication management in patients with psychosis and schizophrenia [29]. As educators and counsellors, mental health nurses address barriers to adherence by using shared decision-making techniques and tools, which engage and empower patients to actively participate in decisions about their treatment [30]. These skills could be applied to improve treatment adherence, medication management, and transitional care in different healthcare settings, such as in patients with psychosis and concomitant HCV infection. The therapeutic relationship that mental health nurses build with these patients and their families is also an advantage to improve both treatment adherence and user satisfaction [31, 32].

A qualitative study developed with patients with HCV who inject drugs revealed the difficulties to access to HCV treatment and highlighted the need for nurse navigator services that help patients navigate through a complex and fragmented healthcare system [33]. Moreover, some experiences with SMD patients with physical comorbidities treated in the US Medicaid system based on a nurse-navigator model have shown promising results in the seamless management of mental and physical health problems [34]. However, for these services to be effective, a solid and consistent approach is required, with well-defined limits and sufficient resolution capacity to bridge the various gaps that arise throughout the process [35]. Ryvicker's [36] behavioral-ecological conceptual framework for navigation addresses these two requirements. Thus, individual predisposing and limiting factors determine the degree to which a patient is able to navigate effectively through the health system (educational level, health literacy, communication skills, degree of self-efficacy, support from family caregivers, among others). The complex interaction between predisposing, facilitating, limiting factors and patient’s needs end up shaping their lifestyles and health practices. Provider factors such as continuity of care from different healthcare professionals, communication or respect and trust towards patients must also be considered. The confluence of these factors influences health outcomes, generating exacerbations of chronic diseases, hospitalizations, changes in functional status or poor quality of life. Finally, this model establishes ecological determinants such as the environment of health services, the social context of the person and the environment of people's lives. In the case of people with SMD both, predisposing and limiting behavioural factors, as well as ecological ones, acquire special relevance. Thus, when a SMD patient is diagnosed with a physical illness and treatment instructions are provided to them, patients will frequently not start the treatment immediately; instead, they will probably wait to discuss it first with their psychiatrist or mental health nurse or go home and not discuss the matter ever again. There are barriers during the consultation process, follow-up, and ongoing care, both associated to the health care providers and the patient that may lead to a poor compliance with a non-psychiatric treatment [37].

## The study

### Aims

The objective of this study is to compare the effectiveness of a nurse navigator program (NNP) developed by a team of mental health nurses, aimed at patients with HCV and SMD versus the usual standard of care. The variables to be assessed include HCV testing, treatment access and adherence, support for navigation throughout the healthcare system, and recovery from HCV.

The null hypothesis of this study is that there will be no differences in the HCV cure rate in SMD subjects who engage in the NNP versus those who receive the standard of care. Additionally, a second hypothesis would be that the intervention improves adherence to HCV treatment, expressed as a percentage based on the taken doses of prescribed treatment during the treatment period (from 0% to ≥ 100%).

### Design/ methods

A randomized clinical trial with blinded outcome assessment. This study has been registered in ClinicalTrials.gov (Identifier code: NCT04891445).

#### Population and sample

##### Population

Patients over 18 years of age included in the SMD program of the Mental Health Unit of the Regional University Hospital of Malaga will participate in the study. This Mental Health Unit is made up of several hospital and community units scattered around the city which will be prioritized to participate in the trial.

Patients for whom, for whatever reason, HCV treatment is contraindicated and patients with active SMD symptoms at the beginning of the study that might influence their ability to take part in the study will be excluded until stabilization. Those who do not give informed consent to participate will also be excluded from the study. In that case, they will receive the standard of care.

##### Sample

As far as we know, there are no comparative studies assessing adherence of SMD patients to HCV treatment. For this reason, adherence to lifelong treatments such as oral antidiabetics in patients with schizophrenia has been taken as a reference. Based on data from Kreyenbuhl et al. [38], in which differences in adherence of patients with schizophrenia reached 10%, with an alpha of 0.05, a beta of 20%, and raising the threshold of differences to 15% because, unlike oral antidiabetics, it is a temporary treatment, the needed sample would be 276, assuming that adherence will be 80% in the intervention group and 65% in the control group. This sample will be increased by 15% to cover losses. Additionally, we considered the adherence threshold obtained by Ho et al. in their pilot non-controlled study for patients with HCV and psychiatric disorders, [39] with the same estimation parameters, resulting in a similar sample size.

##### Endpoints and measures

The cure rate and adherence to treatment will be the primary endpoints. To assess cure, HCV viral load will be determined 8–12 weeks after the end of treatment. Patient adherence to treatment will be evaluated using the AIDS Clinical Trials Group method. By this method, patients are inquired about medications not taken in a period of four days prior to the interview: % adherence = (total galenic units prescribed for that period—total units not taken) / total galenic units prescribed for that period [40]. The cut point for this measure has been set to 95%, as the best threshold for a predictive positive virologic response in patients with HIV [41]. A record will also be kept of whether the patient needed to use a pillbox to ensure medication intake and/or required monitoring by the family or the mental health unit to guarantee the correct use of the treatment.

Secondary endpoints include sociodemographic and clinical data and data related to the healthcare process. Other variables in relation to the patient include changes in daily functioning as assessed using the Life Skill Profile (LSP) [42]; negative symptoms, measured on the Positive and Negative Syndrome Scale (PANSS) [43]; and changes in quality of life, measured with the Euroqol5D Health Questionnaire [44], all of them in their Spanish version (See Table 1). These questionnaires will be administered by an evaluator (psychologist or nurse) trained for it and blind to the experimental condition assigned to each patient.

**Table 1:** Psychometric characteristics of the instruments included

Questionnaire	Domain (s)	Psychometric properties
**Life Skill Profile (LSP)** [42]	Self-care	Concurrent validity, this scale was compared with the social behaviour schedule SBS: 0.56 Interrater reliability *r* = 0.62, and internal consistency α = 0.82
	Interpersonal social behaviour	Concurrent validity, this scale was compared with the social behaviour schedule SBS: 0.54 Interrater reliability *r* = 0.69, and internal consistency α = 0.77
	Communication and social contact	Concurrent validity, this scale was compared with the social behaviour schedule SBS: 0.63 Interrater reliability *r* = 0.86, and internal consistency α = 0.86
	Non-personal social behaviour	Concurrent validity, this scale was compared with the social behaviour schedule SBS: 0.36 Interrater reliability *r* = 0.76, and internal consistency α = 0.78
	Autonomous life	Concurrent validity, this scale was compared with the social behaviour schedule SBS: 0.46 Interrater reliability *r* = 0.76, and internal consistency α = 0.80
**Positive and Negative Syndrome Scale (PANSS)** [43]	Positive symptoms	Concurrent validity, it was compared with the Andreasen positive scale, *r* = 0.70 Interrater reliability ICC = 0.72, and internal consistency α = 0.62
	Negative symptoms	Concurrent validity, it was compared with the Andreasen negative scale, *r* = 0. 81 Interrater reliability ICC = 0.80, and internal consistency α = 0.92
	General psychopathology	Interrater reliability ICC = 0.56, and internal consistency α = 0.55
**EuroQol 5D** [45]	Mobility Self-care Usual activities Pain/ Discomfort Anxiety/Depression	Concurrent validity, it was compared with the SF-6D, *r* = 0.73 [46] Construct validity it was evaluated in spanish population

Sociodemographic (age, sex) and socioeconomic data (job performance), where applicable, will be assessed using the Social Class Classification of the Spanish Society of Epidemiology (if the patient does not have a job, it will be recorded whether the patient is in a situation of retirement, unemployment, temporary disability, or job search). Consumption of drugs of abuse, regular psychiatric treatment, physical comorbidities, and their treatment, as well as type of diagnosis, time from disorder onset, availability of family support, and mental health hospital admissions in the last 12 months will also be recorded. Other data to be collected include adverse events or interactions reported by the patient or family caregiver and other important observations collected during evaluation and follow-up visits.

Regarding healthcare process indicators, the number of previously detected but untreated cases, detection rate after screening the SMD population, percentage of detected cases referred to the digestive service, and delay time until first consultation with the specialist will also be recorded. In addition, scheduled and unscheduled visits led by the monitoring team and number of calls made and received by this team will be recorded. Regarding hepatitis, case classification by genotype and treatment administered will also be recorded. In addition, the prevalence of undiagnosed HCV infection and diagnosed but untreated HCV infection in SMD patients will also be estimated. Finally, a comparison of daily life functioning, occurrence of symptoms, quality of life, and frequency of adverse events during active infection and once cured will be performed.

#### Data collection

Screening for HCV will be performed at the annual somatic control of SMD patients performed by mental health nurses. In the case of the Hospitalization Unit, HCV-positive cases will be extracted from hospital admission records. Detection will be done through blood tests including HCV testing. In case the virus is detected, part of the same blood sample will be used to perform the viral load test. Verbal consent will be requested from the patient or their legal representative prior to laboratory analysis. The patient will be informed that, in the event of a positive HCV and viral load test, they will be offered to participate in the study.

Patients will be randomized using a computer-generated sequence hidden from study participants and only known to a team member who will not participate in sample collection. Subjects will be included sequentially and assigned to the corresponding group through an online consultation system each time a new participant is ready to enter.

#### Interventions

In this study, a navigation program led by mental health nurses and auxiliary nurse technicians from a mental health unit will be implemented.

The control group will receive the standard of care. Patients in this group will be directly referred to their family physician with a report showing a positive HCV and viral load test for prescription of the standard treatment established by the Andalusian Health System. Standard management involves referral to the gastroenterologist, with a significant delay. Generally, the gastroenterologist prescribes a series of tests to confirm diagnosis. These tests will be performed in other units, for which previous appointment is required. Once test results become available, the patient will have another consultation with the gastroenterologist, who will finally establish a diagnosis and prescribe the treatment. The prescribed drug will be dispensed at the hospital pharmacy once a month until the treatment is completed, and cure is confirmed. The last phase of the treatment involves a final visit with the gastroenterologist to confirm that the viral load has disappeared according to a laboratory test.

In the intervention group, patients will be accompanied throughout the evaluation and treatment process until they are fully cured. The details of the intervention are described following the template for intervention description and replication (TIDiER). Follow-up will include a simplification of the clinical pathway of treatment. This way, the mental health service refers the patient to a first visit with the gastroenterologist. To guarantee compliance, all patients will be accompanied to that appointment either by a competent available family caregiver or by a member of the NNP team.

In the first visit, the gastroenterologist will perform a liver ultrasound and determine the most appropriate treatment for each patient. In the same visit, the patient will receive the prescribed pharmacological treatment, which will be dispensed at the hospital pharmacy. Thus, the patient obtains a final diagnosis and his pharmacological treatment the same day following a single-step clinical pathway.

The mental health nurse team will subsequently arrange for the analysis of the genotype of the virus in the mental health service, if necessary.

**Fig. 1:**
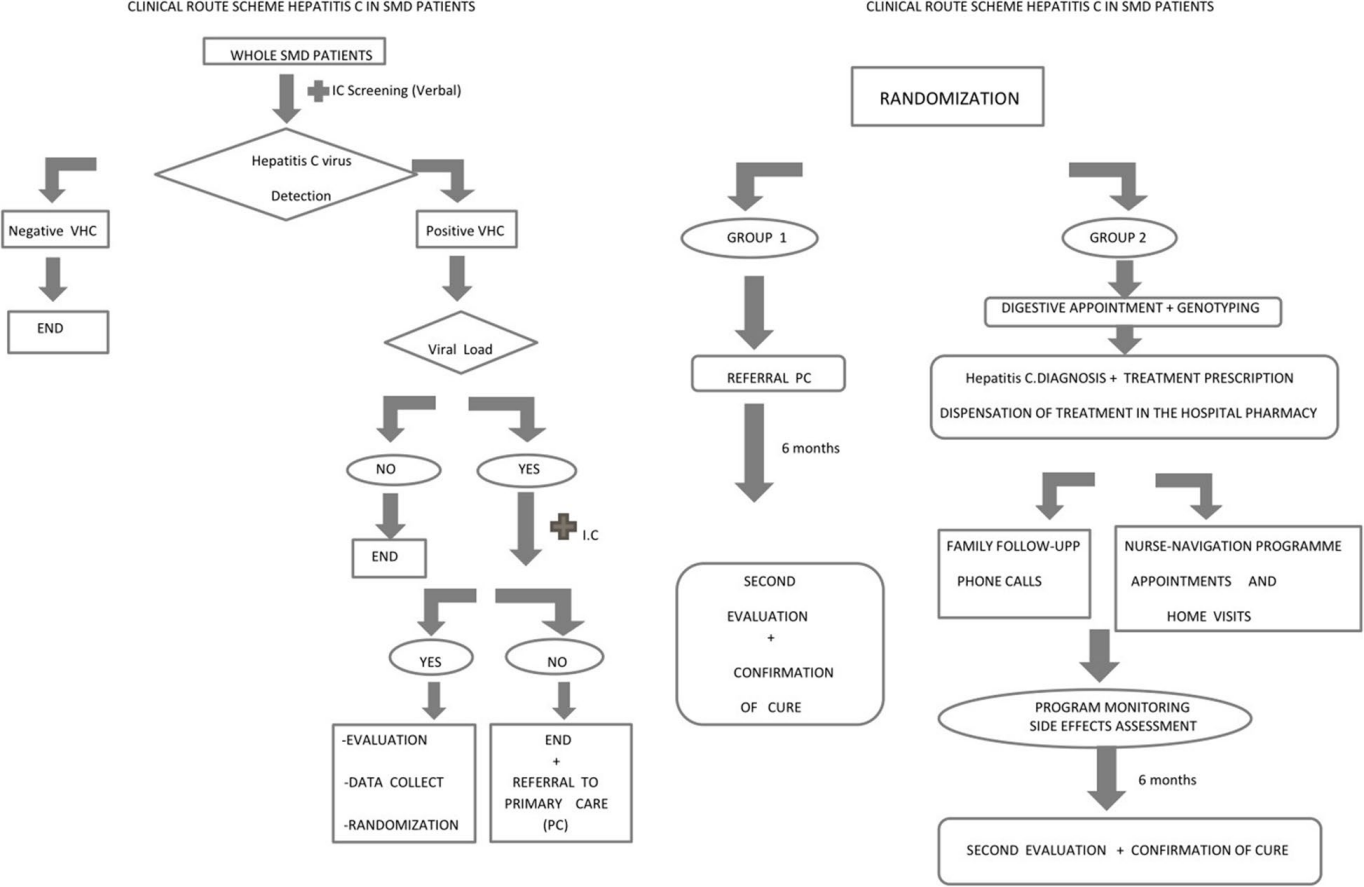
Clinical route scheme hepatitis C in SMD patient

The intervention is based on an *ad-hoc* decision algorithm developed by all the healthcare services involved (See Fig. 1).

#### Monitoring

During the treatment period (8–12 weeks depending on the prescribed drug), actions will be taken to ensure correct treatment adherence. To such purpose, the mental health nurse will assess patient’s ability to comply with the treatment. In case of patient’s inability, the nurse will assess family’s commitment to supervise and remind the patient about drug intake. In both cases, the nurse will carry out an educational intervention to ensure that the patient and/or their family clearly understand treatment guidelines. This intervention is structured through a standardized care plan. In cases in which proper treatment use is not guaranteed, medication will be provided weekly by the monitoring team.

Upon completion of the 8–12 weeks treatment period, a final evaluation of all patients will be performed. The data collected in this evaluation visit include adherence to treatment, person responsible for treatment administration, occurrence of adverse effects or interactions, and number of visits and calls during the treatment period.

A third and final analysis of all study variables will be carried out six months after starting the treatment. Finally, disappearance of the viral load and, therefore, cure from HCV will be determined by a new blood test.

#### Ethics

This study was approved by the Malaga Provincial Research Ethics Committee with the code CQG-20 (0444-N-20). This study will comply with best clinical practice guidelines and the ethical standards for research involving human participants established in the Declaration of Helsinki and its subsequent versions.

Clinical data will be processed in accordance with current legislation on data protection.

In the preliminary phase of the study, consisting of a screening of the entire population diagnosed with a serious mental disorder, only verbal consent is being requested. This blood analysis has been included as part of the annual physical health review offered to patients with severe mental disorders in the Unit, which already involves blood extraction, and the Ethics Committee has approved that written consent is not required when treating of a screening of the entire population.

However, people who finally have a positive viral load (which will predictably be a small percentage of the previous ones) will be offered to participate in the clinical trial, for which they must give their informed consent in writing.

Prior to inclusion in the trial, each subject will be informed in writing and orally of the objectives and methodology of the project. Informed consent will be required for inclusion. If the patient is not legally competent, informed consent from the legal guardian will be required by nursing staff.

Adequate medical assistance will be provided in case patients experienced any adverse event. All patients will be informed of the need for medical assistance for concomitant illnesses of which the research team is aware.

The occurrence of adverse events will be monitored during the study by the Gastroenterology Service (Ward) as part of routine monitoring of HCV patients, in accordance with the effects described in the technical sheet of the medication prescribed to each patient.

In case of occurrence of a serious adverse event, expected or not, related or not to the study, the study will be temporarily suspended. Then, the event will be reported to the Ethics Committee and an audit procedure of the case will be arbitrated to determine the causal chain of the event and the possible factors involved. Urgent measures will be adopted to guarantee patient safety. If it is determined that the event is not related to the study, the latter will be resumed following Ethics Committee authorization. Otherwise, the study will be permanently suspended.

#### Data analysis

First, an exploratory data analysis will be performed to determine the distributions of the different variables and evaluate their normality using the Kolmogorov–Smirnov test, kurtosis analysis and skewness. Measures of central tendency and dispersion will be calculated, as well as percentage distributions. Subsequently, a bivariate analysis will be performed using the Chi-square test for comparison of qualitative variables, and Student's *t*-test for independent groups for quantitative variables. In case of non-parametric distribution, the Mann–Whitney *U* test will be performed.

In addition, repeated measures ANOVA will be used to determine intra-group progression of the different variables throughout the monitoring period, to evaluate the time effect.

Finally, a multivariate regression model will be constructed using Cox regression to evaluate the primary endpoints (cure and adherence), with follow-up periods as time adjusted for the variables found to have a significant association on bivariate analysis. The raw and adjusted hazard ratio for treatment failure and non-adherence will be estimated.

All the analyses will be performed by intention to treat (ITT) and by multiple imputation using the monotonic approach if missing data show a monotonic pattern; otherwise, the fully conditional specification will be used. Patients will be analysed in the group to which they will be initially allocated, regardless of whether they finally receive the randomized intervention.

All analyses will be done with estimation of 95% confidence intervals.

## Discussion

The present study addresses several health issues that have not yet been analysed in Spain. Novel drugs different from interferon with proven effectiveness and tolerability in SMD patients have been approved [47]; the scientific literature, however, still shows that this population is at an increased risk for acquiring infectious diseases such as HCV [17, 48]. There is also evidence of difficulties in detecting and treating HCV and concomitant physical comorbidities in this population [49–51]. This study will provide data on the feasibility of a NNP aimed at improving coordination between the different specialists who treat patients with SMD and physical comorbidities, in this case, HCV. Moreover, if the results show that this approach is effective, it would be applicable to many other comorbidities that are frequently found in this population and remain undiagnosed and undertreated.

Likewise, there is consistent evidence that patients with SMD often have treatment adherence problems [52, 53]. This population of patients also show difficulties in navigating through complex clinical pathways that imply attending several appointments in various health services at different times, probably due to their impaired executive function [54, 55]. In the case of HCV, the clinical pathway involves, but not limited to, visits to the services of mental health, microbiology, primary care, digestive medicine, and hospital pharmacy. When the patient has adherence problems, it may be necessary that they are accompanied throughout complex processes including follow-up visits related to physical health problems. These patients are frequently accompanied by nurses [56, 57]. For this randomized controlled trial, an approach tailored to patient’s needs and context will be adopted to maximize treatment adherence and, as far as possible, ensure resolution of HCV infection. Once the study is completed, this model of care will be offered to the rest of patients of the Mental Health Service Unit of the University Regional Hospital of Málaga. If the trial proves to be effective, this model could be easily applied at other Mental Health services. Moreover, the study is designed from a pragmatic approach, with no extraordinary modifications of daily practice, except for the implementation of an NNP. Thus, the intervention will be implemented by the Mental Health Unit staff itself. If results are satisfactory, the sustainability of the model is guaranteed and its generalization to other Mental Health Units of the Public Health Care System is easily achievable in Spain. Furthermore, mental health services are well-positioned to lead a more active role in the treatment of HCV and engage patients in the treatment process [49].

A secondary contribution of this study is that it will provide data on the prevalence of HCV symptoms such as apathy, anhedonia, tiredness (Negative symptoms scale, PANSS), which are commonly attributed to schizophrenia or to the side effects of usual pharmacological treatments such as antipsychotics or mood stabilizers. During the study, all these symptoms will be recorded at baseline and once infection has been cured. Consequently, it is a unique opportunity to assess the occurrence of symptoms once HCV infection is resolved. If these symptoms persist after HCV infection is controlled, a psychotic aetiology will be confirmed.

### Limitations

This study has several limitations. One of them is that all participants will be recruited in a single hospital; therefore, these findings will require replication in other settings to confirm consistency. Another possible limitation is that there are people with SMD and HCV infection who are not being treated in mental health services (homeless people with psychosis, people with poor accessibility to mental health services, etc.). To overcome this issue, members of several community SMD organizations and family associations will also be recruited.

## Conclusions

The results of this study may demonstrate the effectiveness of a novel NNP in optimizing treatment adherence and ensuring healing from HCV in this population.

## Data Availability

Not applicable.

## Abbreviations

SMD: Severe Mental Disorder
HIV: Human Immunodeficiency Virus
HBV: Hepatitis B virus
HCV: Hepatitis C virus
NNP: Nurse Navigator Program
LSP: Life Skill Profile
PANSS: Positive and Negative Syndrome Scale
ITT: Intention To Treat
